# Utility of Second-Generation Line Probe Assay (Hain MTBDR*plus*) Directly on 2-Month Sputum Specimens for Monitoring Tuberculosis Treatment Response

**DOI:** 10.1128/JCM.00025-17

**Published:** 2017-04-25

**Authors:** Neesha Rockwood, Justyna Wojno, Yonas Ghebrekristos, Mark P. Nicol, Graeme Meintjes, Robert J. Wilkinson

**Affiliations:** a Department of Medicine, Imperial College, London, United Kingdom; b Clinical Infectious Diseases Research Initiative, Institute of Infectious Disease and Molecular Medicine, University of Cape Town, Cape Town, Republic of South Africa; c National Health Laboratory Service, Groote Schuur Hospital, Cape Town, Republic of South Africa; d Division of Microbiology, Department of Pathology, University of Cape Town, Cape Town, Republic of South Africa; e Department of Medicine, University of Cape Town, Cape Town, Republic of South Africa; f The Francis Crick Institute, London, United Kingdom; Carter BloodCare & Baylor University Medical Center

**Keywords:** line probe assay, treatment outcome, drug resistance, bacterial biomarker, tuberculosis

## Abstract

The utility of a line probe assay (Genotype MTBDR*plus*) performed directly on 2-month sputa to monitor tuberculosis treatment response is unknown. We assessed if direct testing of 2-month sputa with MTBDR*plus* can predict 2-month culture conversion and long-term treatment outcome. Xpert MTB/RIF-confirmed rifampin-susceptible tuberculosis cases were recruited at tuberculosis diagnosis and followed up at 2 and 5 to 6 months. MTBDR*plus* was performed directly on 2-month sputa and on all positive cultured isolates at 2 and 5 to 6 months. We also investigated the association of a positive direct MTBDR*plus* at 2 months with subsequent unsuccessful tuberculosis treatment outcome (failure/death during treatment or subsequent disease recurrence). A total of 279 patients (62% of whom were HIV-1 coinfected) were recruited. Direct MTBDR*plus* at 2 months had a sensitivity of 78% (95% confidence interval [CI], 65 to 87) and specificity of 80% (95% CI, 74 to 84) to predict culture positivity at 2 months with a high negative predictive value of 93% (95% CI, 89 to 96). Inconclusive genotypic susceptibility results for both rifampin and isoniazid were seen in 26% of MTBDR*plus* tests performed directly on sputum. Compared to a reference of MTBDR*plus* performed on positive cultures, the false-positive resistance rate for direct testing of MTBDR*plus* on sputa was 4% for rifampin and 2% for isoniazid. While a positive 2-month smear was not significantly associated with an unsuccessful treatment outcome (adjusted odds ratio [aOR], 2.69; 95% CI, 0.88 to 8.21), a positive direct MTBDR*plus* at 2 months was associated with an unsuccessful outcome (aOR 2.87; 95% CI, 1.11 to 7.42). There is moderate utility of direct 2-month MTBDR*plus* to predict culture conversion at 2 months and also to predict an unfavorable outcome.

## INTRODUCTION

As part of the global strategy to diagnose, treat, and reduce the transmission of drug-sensitive (DS) and drug-resistant (DR) tuberculosis (TB), molecular diagnostic tests have reduced time to treatment (1). Interim outcome measures such as 2-month culture conversion have merit as a surrogate of long-term treatment outcomes and hence may expedite progression to testing of novel regimens in clinical trial settings (2). The good negative predictive value of a negative culture at 2 months for the long-term treatment outcomes of failure/relapse can be used by clinicians as a marker of treatment response, with a subsequent switch from a quadruple-drug regimen to dual therapy (3). Drug pressure may select for mutations conferring resistance or amplify resistance during treatment. This process can occur at different time points during treatment, ranging from within the first 2 months, when the bacterial load is highest, to later in treatment, and this can be screened for by using molecular or culture-based drug susceptibility tests on sputum obtained during treatment. Such screening is typically conducted at 2 months and at month 5 or 6 of treatment (at which point the patient is considered to have failed treatment if still culture positive) (4). Acquired drug resistance (ADR) is influenced by baseline drug susceptibility patterns, HIV-1 coinfection, baseline extent of disease, and treatment adherence (5).

The GenoType MTBDR*plus* line probe assay (LPA), version 2.0 (Hain Lifescience, Nehren, Germany), is a molecular diagnostic assay that has been widely used to identify resistance to first-line drugs rifampin and isoniazid among TB cases. It uses PCR to amplify regions specific to Mycobacterium tuberculosis complex, areas of the *rpoB* gene associated with rifampin resistance, and *katG* and *inhA* genes associated isoniazid resistance from smear-positive or smear-negative clinical samples (6, 7) or cultured isolates. Hybridization of amplified sequences to a membrane strip (with immobilized probes for both wild-type and mutant sequences) identifies specific mutant bands or the absence of wild-type bands. It appears to be more sensitive than Xpert MTB/RIF to detect heteroresistance, defined as the simultaneous presence of DS and DR populations in the same patient, thought likely to be an early step in the pathway of resistance amplification (8). ADR can be reliably detected only when drug-resistant mutants are present at greater than 65% and 5% of the overall bacterial population for GeneXpert MTB/RIF (9) and MTBDR*plus*, respectively (8, 10). Limitations of both tests in treatment monitoring include amplification of DNA from nonviable bacteria.

We prospectively determined the utility of MTBDR*plus* to predict culture conversion when carried out directly on a 2-month sputum specimen. We also assessed the potential of a direct MTBDR*plus* to detect early ADR/heteroresistance on a 2-month sputum specimen, compared with a reference of MTBDR*plus* on positive 2-month cultured isolates. We also determined the association between a positive 2-month MTBDR*plus* result and the composite long-term unsuccessful outcome of failure/death during treatment or TB recurrence.

## RESULTS

### Outcomes.

From the larger study of 306 participants, overall 279 participants (62% HIV coinfected, 51% with smear grade 1+ to 3+ at baseline, and 32% of retreatment status), who had a direct MTBDR*plus* test carried out on 2-month sputum, were included in the 2-month culture conversion analyses. Patients in the cohort had clinical follow-up for the duration of TB treatment and a follow-up at a median of 22 months (interquartile ratio [IQR], 18 to 28) to ascertain disease recurrence via electronic database searches. The smear and culture conversion rates at 2 months were 88% and 79%, respectively. There were no culture-confirmed cases of ADR at 2 months. At 5 to 6 months, the rate of culture conversion (the denominator including only those who produced sputum at 5 to 6 months) was 223/233 (95%). The culture conversion/treatment completion rate was 256/279 (92%). There were 267/279 participants who had both a 2-month direct MTBDR*plus* result and a known long-term treatment outcome. There were 8 individuals with a negative 2-month MTBDR*plus* result who defaulted on treatment and 3 who were lost to follow-up. There was 1 individual with a positive 2-month direct MTBDR*plus* result who had an unknown long-term treatment outcome. Two cases of ADR (1 with acquired isoniazid resistance and 1 with acquired rifampin resistance) were identified on MTBDR*plus* and phenotypic drug susceptibility testing (DST) of positive 5- to 6-month cultures. Overall, there were 21/267 (8%) individuals with unsuccessful outcomes (11 failures/deaths during treatment and 10 TB recurrences (see Fig. 2; see also Table S1 in the supplemental material).

### Direct MTBDR*plus* for assessment for culture conversion at 2 months.

A positive direct MTBDR*plus* on a 2-month sputum sample (presence of M. tuberculosis complex band [TUB band], amplification and locus control) had a sensitivity of 78% (95% CI, 65 to 87) and specificity of 80% (95% CI, 74 to 85) to predict positivity at 2 months in liquid (MGIT) cultures. The negative predictive value of direct MTBDR*plus* on 2-month sputa to predict culture conversion was high, 93% (95% CI, 89 to 96) (Table 1). The sensitivity and specificity of a positive smear test carried out on the 2-month sample were 41% (95% CI, 29 to 55) and 96% (95% CI, 92 to 98), with a negative predictive value of 86% (95% CI, 81 to 90). The proportion of positive direct MTBDR*plus* results at 2 months was significantly higher in HIV-1-uninfected patients than in HIV-1-infected patients (*P* = 0.04), in patients with baseline smear grades 1+ to 3+, in smear-scanty/negative (*P* < 0.01) and retreatment patients, and in new patients (*P* = 0.04) (Table 2). However, when stratified by HIV-1 serostatus, there were no significant differences in sensitivity, specificity, or positive or negative predictive value of the direct MTBDR*plus* to predict 2-month culture conversion (Table 1). There were no associated clinical characteristics noted in the 13 individuals who were direct MTBDR*plus* negative and culture positive for whom a negative result did not correctly predict culture conversion at 2 months.

**TABLE 1:** Utility of MTBDR*plus* directly on 2-month sputa to detect 2-month culture positivity^*a*^

Assay characteristic	No. for MTBDR*plus* directly on 2-month sputa/no. for reference method (detection of culturable M. tuberculosis in liquid culture in MGIT) (%, 95% CI)		
	All (*n* = 279)	HIV infected (*n* = 172)	HIV uninfected (*n* = 107)
Sensitivity	45/58 (78, 65–87)	23/30 (77, 58–90)	22/28 (79, 59–92)
Specificity	177/221 (80, 74–85)	118/142 (83, 76–89)	59/79 (75, 64–84)
Positive predictive value	45/89 (51, 40–61)	23/47 (49, 34–64)	22/42 (52, 36–58)
Negative predictive value	177/190 (93, 89–96)	118/125 (94, 89–98)	59/65 (90, 81–97)

a Comparison of characteristics of direct MTBDR*plus* assay using 2-month sputa (for predicting 2-month culture conversion) and of reference method (liquid culture in MGIT), with stratification of the cohort by HIV-1 serostatus to assess the effect of HIV-1 serostatus on the utility of the test.

**TABLE 2:** Direct MTBDR*plus* results at 2 months stratified by HIV-1 serostatus, baseline smear grade, and retreatment status

Direct MTBDR*plus*-positive results for M. tuberculosis complex (*n* = 89)								
No. (%) of patients:		*P* value	No. (%) of patients with baseline smear grade:		*P* value	No. (%) of patients who were:		*P* value
Positive for HIV-1 infection (total *n* = 172)	Negative for HIV-1 infection (total *n* = 107)		Negative/scanty (total *n* = 136)	1+ to 3+ (total *n* = 43)		New (total *n* = 189)	In retreatment (total *n* = 90)	
47 (27)	42 (39)	0.04	27 (20)	62 (43)	<0.01	53 (28)	36 (40)	0.04

### Direct MTBDR*plus* for assessment of long-term treatment outcomes.

The 2-month direct MTBDR*plus* test was more likely to be positive in those with unsuccessful outcomes, 12/21 (57%), than in those with successful outcomes, 76/246 (31%) (*P* = 0.01). The 2-month smear positivity rates were also significantly increased in those with unsuccessful outcomes, 6/21 (29%) compared with those with successful outcomes, 27/246 (11%) (*P* = 0.02). There was no significant difference in the proportion of unsuccessful treatment outcomes for new versus retreatment patients (see Table S2 in the supplemental material).

When adjusted for baseline extensive radiological disease and HIV-1 serostatus, a positive direct MTBDR*plus* at 2 months (adjusted odds ratio [aOR], 2.88; 95% CI, 1.11 to 7.42). A positive 2-month smear was not significantly associated with an unsuccessful treatment outcome (aOR, 2.69; 95% CI, 0.88 to 8.21).

### Direct MTBDR*plus* for assessment of ADR.

The use of MTBDR*plus* directly on 2-month sputa did not identify any culture-proven cases of ADR. There were no cases in which both wild-type and mutant bands were simultaneously represented on the MTBDR*plus* strip, demonstrating heteroresistance. There were inconclusive rifampin and isoniazid genotypic susceptibility results in 26% of positive direct MTBDR*plus* tests. When this analysis was restricted to the 2-month samples that subsequently had paired positive cultures at 2 months, the proportions of inconclusive rifampin and isoniazid genotypic susceptibility results were reduced to 16% and 7%, respectively (Table 3). There were false rifampin resistance calls in 4% of cases and false isoniazid resistance calls in 2% of cases (Table 4). None of these cases showed heteroresistance on review of the MTBDR*plus* membrane strip. On repeat direct MTBDR*plus* tests performed on archived sputa, the result was either negative for MTB complex or rifampin/isoniazid susceptible. Only 3 sputa (of 11 with false-positive rifampin with or without isoniazid resistance) were subsequently culture positive, and DST on the culture showed rifampin/isoniazid susceptibility. All patients with false-positive resistance results completed standard anti-TB treatment and had successful treatment outcomes.

**TABLE 3:** Utility of MTBDR*plus* directly on 2-month sputum to detect acquired drug resistance: inconclusive results

MTBDR*plus* result^*a*^ direct on 2-mo sputa	No. (%) of results deemed positive (of a total of 89 samples)	No. (%) restricted to confirmatory positive culture^*b*^ (of a total of 58 samples)
Inconclusive INH genotypic susceptibility result	23 (26)	4 (7)
Inconclusive RIF genotypic susceptibility result	23 (26)	9 (16)

a INH, isoniazid; RIF, rifampin.

b All positive cultures had confirmed rifampin and isoniazid susceptibilities.

**TABLE 4:** Utility of MTBDR*plus* directly on 2-month sputum to detect acquired drug resistance: false-positive results

MTBDR*plus* result^*a*^	No. (%) of samples (total *n* = 279)	Repeat direct MTBDR*plus* test results^*b*^
False-positive isoniazid resistance	6 (2)	Result was negative for MTB complex in 4/6 cases and isoniazid susceptible in 2/6 cases
False-positive rifampin resistance	11 (4)	Result was negative for MTB complex in 7/11 cases and rifampin susceptible in 4/11 cases

a Compared with MTBDR*plus* and phenotypic drug susceptibility testing performed on culture if flagged positive. Only 3 patients (of 11 with false-positive rifampin with or without isoniazid resistance) were subsequently culture positive.

b On archived sputa in cases of suspected false-positive resistance.

## DISCUSSION

This is the first study to report the utility of MTBDR*plus* directly on sputum to predict 2-month positivity in liquid culture. The sensitivity was superior to that of a 2-month smear (78% versus 41%), and the specificity was inferior (80% versus 96%). The suboptimal sensitivity and positive predictive value of MDTBR*plus* directly on sputum do not support substitution of this assay for the conventional culture method to monitor interim treatment response.

Friedrich et al. assessed the utility of Xpert/MTB RIF (also carried out directly on 2-month sputum samples) to monitor treatment response against conventional smear and culture positivity in 177 HIV-1-uninfected patients. They reported a sensitivity and specificity of 95% (95% CI, 87 to 98) and 23% (95% CI, 15 to 32), respectively, for predicting 2-month culture positivity with a positive predictive value of 47% (95% CI, 39 to 56) and a negative predictive value of 85% (95% CI, 66 to 96) (11). Hence, the specificity of the direct MTBDR*plus* assay (80%) was much better than that of the results reported for the qualitative Xpert MTB/RIF test. This was not explained by potential differences in sputum bacillary load in HIV-1-infected and -uninfected individuals (12) (Friedrich et al. excluded HIV-coinfected individuals, whereas 62% of individuals in this study were HIV-1 coinfected). A negative MTBDR*plus* test had a high negative predictive value for 2-month culture conversion in both HIV-1-infected and HIV-1-uninfected individuals. There was a significantly lower sensitivity for culture positivity of direct MTBDR*plus* than for Xpert MTB/RIF at 2 months (78% versus 95%). Potentially, the former is less likely to be falsely positive (secondary to DNA from nonviable bacilli) several months following successful treatment of TB than has been reported with Xpert MTB/RIF (13). However, this hypothesis should be confirmed in a study directly comparing Xpert MTB/RIF and MTBDR*plus* for the same samples.

A study by Shenai et al. showed that by using Xpert MTB/RIF cycle threshold (*C_T_*) parameters to estimate bacterial load, a *C_T_* of 28.6 at month 2 of chemotherapy had a sensitivity of 88.6% (95% CI, 77 to 95) and a specificity of 76.3% (95% CI, 60 to 89%) for prediction of culture positivity. These results are close to our results obtained with MTBDR*plus* directly on sputum (14).

Preemptive detection of heteroresistance, which may give rise to ADR, can be challenging. While phenotypic or genotypic DST on a positive culture is currently the reference standard, it can take several weeks to culture M. tuberculosis from expectorated sputum, delaying the commencement of individualized treatment regimens in cases of ADR. In this study, neither of the cases of ADR detected by MTBDR*plus* and phenotypic DST at 5 to 6 months was detected as heteroresistance on direct MTBDR*plus* at 2 months. The majority of sputa at 2 months are smear negative, and this study demonstrated unacceptably high rates of inconclusive and false-positive resistance results using MTBDR*plus* directly on sputum compared to culture as the gold standard. The inconclusive results may be secondary to insufficient DNA in the context of increased amplification cycles included in the direct MTBDR*plus* test protocol. This interpretation is supported by the lower proportion of inconclusive direct MTBDR*plus* genotypic susceptibility results from samples that were culture positive versus culture negative. The false-positive resistance results may be secondary to handling PCR amplicons in open molecular diagnostic laboratories dealing with high volumes of routine clinical samples. Repeat direct MTBDR*plus* results for 2-month archived sputa from patients who had false-positive resistance results either were negative or showed rifampin/isoniazid susceptibility. Only 3/11 patients who had false-positive resistance results on direct MTBDR*plus* at 2 months were subsequently culture positive, and DST on these cultures also showed RIF/INH susceptibility.

Although this finding was not the primary aim of the study, in this cohort, a positive MTBDR*plus* at 2 months was associated with an approximately 3-fold-increased odds of the composite unsuccessful outcome of death/failure/relapse, having adjusted for baseline extensive disease on chest radiograph and HIV-1 status. MTBDR*plus* was better than 2-month smear at predicting unsuccessful outcomes.

A strength of this study was that the same 2-month sputum specimen was utilized for carrying out both direct MTBDR*plus* testing and liquid culture. This reduced the variability associated with cough efforts and M. tuberculosis inoculum size. A limitation of this study is that direct MTBDR*plus* testing was carried out at only a single time point during treatment, thus limiting its assessment as a biomarker of treatment response earlier or later in treatment. Archived sputa, used to repeat direct MTBDR*plus* tests for patients with false-positive resistance results, were frozen, and a smaller volume than the original sample was tested. Hence, this may not have been an ideal comparison. There was no clear explanation for the 13 individuals who were direct MTBDR*plus* negative and culture positive at 2 months. It is possible that the bacillary load was simply too low. In this cohort, there were only 2 culture-confirmed cases of ADR, neither of which were detected at 2 months by direct MTBDR*plus*. Hence, we were limited in our capacity to assess the ability of direct MTBDR*plus* as a screening tool for ADR. However, the inconclusive and false-positive genotypic resistance results are clearly of concern.

### Conclusion.

In conclusion, the direct MTBDR*plus* may have moderate value in predicting 2-month culture conversion, its major value being that if negative, there is a 93% probability that the 2-month culture will be negative. Its potential as a biomarker of long-term treatment outcomes warrants further research. Use of direct MTBDR*plus* for routine treatment monitoring is not recommended. Its use in patient management should be carried out with caution, and the diagnostic utility and limitations should be interpreted in the appropriate clinical context.

## MATERIALS AND METHODS

### Setting.

This study was carried out as part of a larger study assessing the frequency and determinants of ADR in patients with baseline rifampin-susceptible pulmonary TB. Participant recruitment was at Site B Ubuntu Clinic, an integrated HIV-1/TB primary care clinic in Khayelitsha Western Cape, South Africa, from March 2013 to July 2014. All laboratory work was carried out at a routine diagnostic laboratory, National Health Laboratory Service (Groote Schuur Hospital), which annually receives approximately 9,000 respiratory samples for TB culture (13% positive). On average, 100 to 130 MTBDR*plus* LPAs are carried out per month.

### Recruitment and procedures.

Participants with Xpert MTB/RIF-confirmed rifampin-sensitive pulmonary TB were recruited at the start of TB therapy. They were excluded if they were less than 18 years of age, refused HIV testing, had received more than 3 doses of treatment, or had received TB treatment in the previous 6 months. Treatment followed local TB program guidelines with 2 months of therapy with rifampin, isoniazid, pyrazinamide, ethambutol (7 days/week) followed by 4 months of rifampin and isoniazid (7 days/week). All participants had HIV-1 serology testing and chest radiography at baseline. Extensive disease on chest X-ray was noted as either involvement of both lungs or involvement of ≥1 of 3 (upper, middle, or lower) zones per lung. The study was approved by the University of Cape Town Human Research Ethics Committee (HREC Ref 568/2012).

At baseline (start of TB treatment) and at 2 and 5 to 6 months, 3 sputa were collected using sputum induction with 3% saline. The largest-volume sputum was sent for culture, and the other 2 samples were frozen and subsequently used for culture in the case of culture contamination. Standard NaOH-N-acetyl-l-cysteine decontamination (final NaOH concentration, 1.5%) and concentration were performed. One or 2 drops obtained directly from the pellet was used for microscopy, and then the pellet was resuspended in 0.5 ml of phosphate-buffered saline (PBS) prior to inoculation in mycobacterial growth indicator tube (MGIT) cultures as per the manufacturer's instruction. MTBDR*plus* LPA, version 2.0, was carried out on all baseline-positive MGIT cultures as per the manufacturer's instructions.

At 2 months, the largest-volume sputum was used for direct microscopy, the direct MTBDR*plus* test, and MGIT culture. The pellet was resuspended in 1 ml PBS, with a 0.5-ml aliquot used for direct MTBDR*plus* and a 0.5-ml aliquot inoculated into MGIT for culture. The manufacturer-recommended MTBDR*plus* protocol included an additional 20 amplification cycles when performed directly on clinical samples, compared with cultured samples. In cases of new isoniazid/rifampin resistance detected by direct MTBDR*plus* at 2 months, another direct MTBDR*plus* test was performed on an archived 2-month sputum from the same patient. All suspected cases of ADR were confirmed via both MTBDR*plus* and phenotypic drug susceptibility testing (DST) carried out on positive culture isolates of the paired 2-month sputum. Phenotypic DST was via the Bactec MGIT 960 system as previously described (15). When the result of MTBDR*plus* performed directly on sputum was internally valid (in terms of all control bands) but did not match the culture DST result, this was considered to be a false-positive result. MTBDR*plus* was also performed on positive cultures at 5 to 6 months of follow-up, with confirmation of cases of ADR with phenotypic DST.

**FIG 1:**
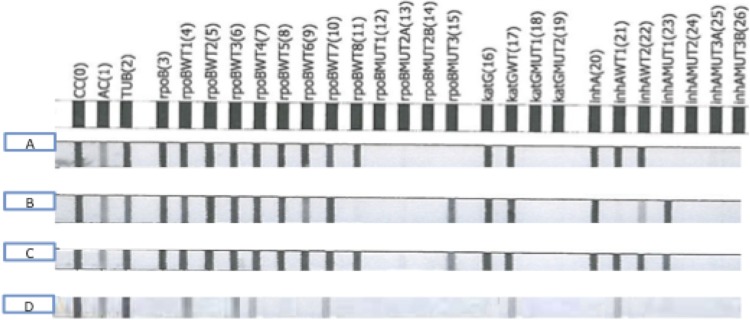
Interpretation of MTBDR*plus* results. (A) Positive for M. tuberculosis complex (presence of TUB band while in the presence of both a conjugate control [CC] and an amplification control [AC]), RIF/INH susceptible (presence of all wild-type bands, absence of all mutation bands). (B) Positive for M. tuberculosis complex (presence of TUB band while in the presence of both a CC and an AC), RIF/INH resistant (absence of wild-type band *rpoB*WT8, presence of mutation band *rpoB*MUT3; absence of wild-type band *inhA*WT1 and presence of mutation band *inhA*MUT1). (C) Positive for M. tuberculosis complex (presence of TUB band while in the presence of both a CC and an AC), RIF heteroresistant, INH heteroresistant (presence of all wild-type bands; presence of mutation band *rpoB*MUT3; presence of mutation band *inhA*MUT1). (D) Positive for M. tuberculosis complex (presence of TUB band while in the presence of both a CC and an AC), inconclusive RIF and INH susceptibility (inconclusive because missing *rpoB*, *inhA*, and *katG* locus control regions [compared with amplification control] and numerous faint/missing wild-type bands).

**FIG 2:**
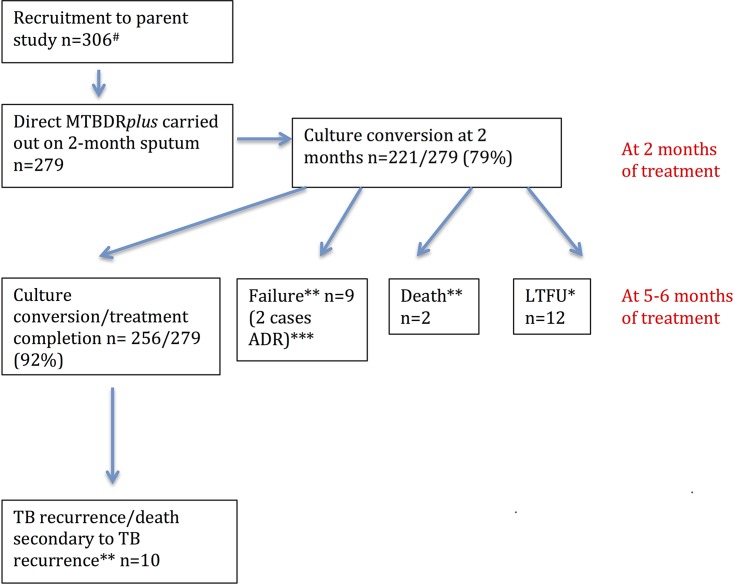
Study outcomes. #, 17 patients did not produce sputa at 2 months, 2 died before follow-up, and 8 produced only 1 sputum, which was erroneously used only for culture; *, 1 LTFU (lost to follow-up) participant had a positive MTBDR*plus* result, and 11 LTFU participants had a negative MTBDR*plus* at 2 months; **, unsuccessful outcomes (*n* = 21); ***, 1 case acquired isoniazid resistance, and 1 case acquired rifampin resistance.

Figure 1 illustrates the interpretation of the MTBDR*plus* line probe assay. Study outcomes are illustrated in Fig. 2.

### Definition of treatment outcomes.

The 2-month culture conversion was defined by a negative 2-month MGIT culture. At 5 to 6 months of treatment, those who were culture negative were defined as cured. Those who were culture positive at 5 to 6 months were defined as treatment failures. Those who completed treatment and were symptom free but without microbiological ascertainment of cure were classified as treatment completers. Electronic database searches of (i) Western Cape Department of Health Data Repository and (ii) National Health Laboratory Service database were conducted to ascertain reported deaths and TB recurrences following completion of study clinical follow-up until the censor date (1 November 2015). “True recurrence” was defined as culture positivity and/or smear 2+/3+ positivity. “Possible recurrence” was defined as positive Xpert MTB/RIF result of a differing resistance profile to baseline and/or a smear grading of scanty/1+ positivity in the absence of culture confirmation.

All treatment failures, deaths during TB treatment, and true/possible TB recurrences were classified as “unsuccessful outcomes.” Deaths subsequent to treatment completion/cure that were not attributable to definite/possible TB recurrence were not defined as unsuccessful outcome. Those who had treatment cure/completion with no microbiologically confirmed TB recurrence (by censor date) were classified as “successful outcome.”

### Statistical analyses.

The two-tailed chi-square test and multivariate logistic regression analysis were carried out in Stata/SE (13.1). In determining the association between a positive 2-month MTBDR*plus* or smear and the composite long-term unsuccessful outcome of failure/death during treatment/TB recurrence, it was elected *a priori* to adjust for the potential confounders HIV-1 serostatus and extensive radiological disease at baseline. Individuals who defaulted on treatment or were lost to follow-up or transferred care with unknown long-term treatment outcome were excluded from long-term treatment outcome analyses.

## Supplementary Material

Supplemental material
