# Supplementary material for: Utility of Second-Generation Line Probe Assay (Hain MTBDRplus) Directly on 2-Month Sputum Specimens for Monitoring Tuberculosis Treatment Response
Source: J Clin Microbiol. 2017 Apr 25;55(5):1508–15. doi: 10.1128/JCM.00025-17 (PMC5405268; doi:10.1128/JCM.00025-17)
Supplement: Supplemental material [file supp_55_5_1508__index.html]

Utility of Second-Generation Line Probe Assay (Hain MTBDRplus) Directly on 2-Month Sputum Specimens for Monitoring Tuberculosis Treatment Response — Supplemental material 

# Utility of Second-Generation Line Probe Assay (Hain MTBDR*plus*) Directly on 2-Month Sputum Specimens for Monitoring Tuberculosis Treatment Response

## Supplemental material

- Supplemental file 1 -

  Tables S1 (Treatment outcomes stratified by 2-month culture converter status) and S2 (Long-term treatment outcomes)

  PDF, 48K
