# Supplementary material for: Utility of Second-Generation Line Probe Assay (Hain MTBDRplus) Directly on 2-Month Sputum Specimens for Monitoring Tuberculosis Treatment Response
Source: J Clin Microbiol. 2017 Apr 25;55(5):1508–15. doi: 10.1128/JCM.00025-17 (PMC5405268; doi:10.1128/JCM.00025-17)
Supplement: Supplemental material [file JCM.00025-17_zjm999095469s1.pdf]

Supplementary table 1 Treatment outcomes stratified by 2-month culture converter status

Outcome	Culture positive at 2 months	Culture negative at 2 months
Failure/death at 5-6 months	5/58 (9%)	6/209 (3%)
Recurrent disease	4/58 (7%)	6/209 (3%)
Loss to follow up	0/58 (0)	12/221 (5%)

Supplementary table 2

	New	Retreatment	p-value
Long term treatment outcome*	11/180 (6%)	10/87 (11%)	0.13

*9 new patients and 3 retreatment patients were not included in the denominator for long term treatment outcome as they were lost to follow up
